# Extracellular Vesicles in Premature Aging and Diseases in Adulthood Due to Developmental Exposures

**DOI:** 10.14336/AD.2021.0322

**Published:** 2021-09-01

**Authors:** Marisa R Pinson, Dae D Chung, Amy M Adams, Chiara Scopice, Elizabeth A Payne, Monisha Sivakumar, Rajesh C Miranda

**Affiliations:** Department of Neuroscience and Experimental Therapeutics, Texas A&M University Health Science Center, Bryan, TX 77807, USA

**Keywords:** extracellular vesicles, developmental origin of health and disease, prenatal exposure, early childhood adversity, miRNA

## Abstract

The developmental origins of health and disease (DOHaD) is a paradigm that links prenatal and early life exposures that occur during crucial periods of development to health outcome and risk of disease later in life. Maternal exposures to stress, some psychoactive drugs and alcohol, and environmental chemicals, among others, may result in functional changes in developing fetal tissues, creating a predisposition for disease in the individual as they age. Extracellular vesicles (EVs) may be mediators of both the immediate effects of exposure during development and early childhood as well as the long-term consequences of exposure that lead to increased risk and disease severity later in life. Given the prevalence of diseases with developmental origins, such as cardiovascular disease, neurodegenerative disorders, osteoporosis, metabolic dysfunction, and cancer, it is important to identify persistent mediators of disease risk. In this review, we take this approach, viewing diseases typically associated with aging in light of early life exposures and discuss the potential role of EVs as mediators of lasting consequences.

## 1. Developmental Origins of Health and Disease

**Figure 1.**
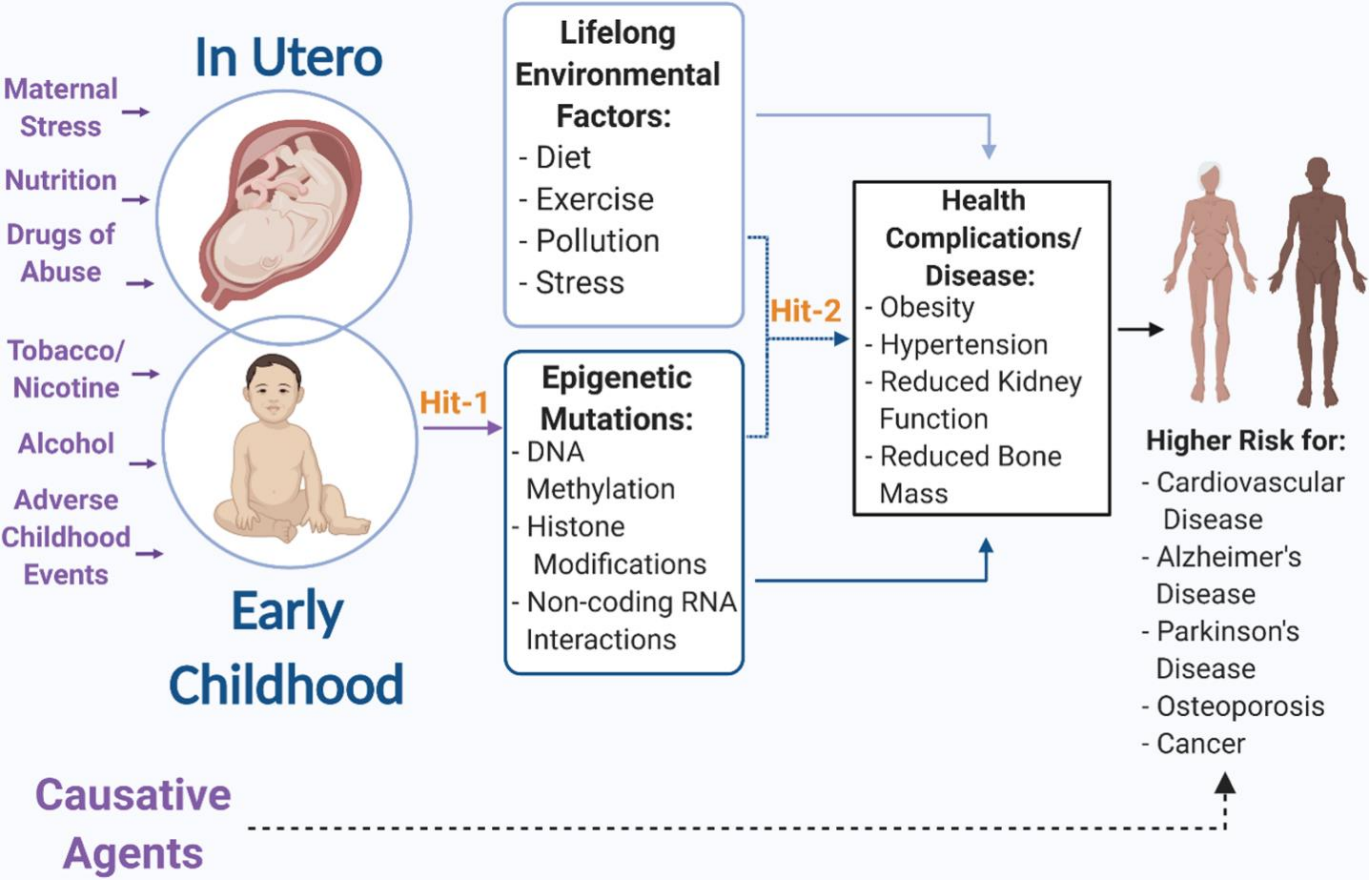
**Early life exposures during pregnancy and childhood act as a first hit that creates lasting epigenetic modifications that increase risk of health complications and diseases later in life**. Additionally, this first hit makes the individual more susceptible to a second environmental hit during life that may further increase risk of disease or increase likelihood of adverse events. (Created with BioRender.com.)

The epidemiologist, David Barker, first presented evidence in the 1980s and 1990s which suggested that maternal/fetal undernutrition at various stages of pregnancy could alter the susceptibility of offspring to metabolic and cardiovascular disease in later life [1, 2]. These data suggested that fetal mis-programming during critical developmental periods could have long-term adverse consequences for adult health. Over time, this fetal programming hypothesis evolved into a generalized paradigm, termed the Developmental Origins of Health and Disease (DOHaD), and a significant area for research focused on identifying mechanisms that mediate the effects of environmental perturbations in early development on health outcomes and disease predisposition in later life (Fig. 1). This area of study has contributed towards the development of a more expansive framework, wherein insults *in utero* or in early life such as undernutrition [1-3], stressors [4], and teratogens, such as alcohol [5-7], may lead to epigenetic modifications with consequences lasting into adulthood. The evolution of the DOHaD framework is coincident with the evolution of a related ‘two-hit’ hypothesis which was originally developed by the cancer biologist Alfred Knudson [8], to explain that retinoblastoma, an ocular cancer in young children, was due to the accumulation of two mutational events or two “hits”. This theory that two or more insults that occur early in development may have synergistic repercussions later in life can be applied to the DOHaD hypothesis. For instance, hypertension [3, 9-11], stroke [12,13], neurodegenerative disorders [14,15], osteoporosis [16,17], sleep disorders [18-25], organ fibrosis [26-28], metabolic dysfunction [29-33] and cancer [34, 35] are all diseases that have an onset in later life, and could be triggered by temporally proximate experiences, but the increased risk for these diseases could have a developmental origin. Furthermore, several disorders better appreciated for their developmental origins, such as Fetal Alcohol Spectrum Disorders (FASDs), are associated with premature aging [5], suggesting that early developmental experiences can influence the onset of diseases that are typically considered aging-related. Here we will briefly describe several examples on how prenatal and early life exposures and stressors have been shown to contribute to impaired health and diseases in later life.

### 1.1 Prenatal alcohol exposure as an initial hit

Fetal Alcohol Spectrum Disorders (FASDs) is a term coined to describe a spectrum of developmental abnormalities that are initiated by prenatal alcohol exposure (PAE) and have the potential to affect multiple organ systems [36, 37]. The most visibly severe end of the spectrum is Fetal Alcohol Syndrome (FAS), which is characterized by, but is not limited to, developmental delays and cranio-facial abnormalities [36]. The estimate for school-aged children affected by FASD is 1.1-5% in the United States [38]. However, in two separate statewide studies in the US, the prevalence of PAE was demonstrated to be higher than previous estimates [39, 40]. With over 50% of pregnancies being unplanned [41], and 22.5% of women consuming alcohol within the first month of pregnancy [41], PAE in an unintentional pregnancy is a likely possibility. The effects of PAE can be viewed with a two-hit hypothesis perspective. The altered programming of genes due to alcohol exposure is the result of the first “hit”, which in turn has been shown to predispose individuals with FASD for negative health outcomes such as cardiovascular disease [42-45], compromised immune system [46-51], and diabetes and metabolic disorders [52-55]. Stressors or environmental factors in later life can subsequently interact with the ethanol-induced genetic modifications, acting as a second “hit” to tip the balance favoring development of early onset systemic diseases. For example, one study reported that both single and repeated maternal ethanol exposures resulted in redistribution of fetal blood flow to support pertinent organs such as the brain [56]. This ‘brain sparing’ phenomena is associated with disproportionate fetal growth [57, 58], and may impede the development of other organs. For example, the number of renal nephrons is permanently reduced by acute alcohol exposure in early development [59]. This reduction in nephron number has also been observed in patients with primary hypertension, suggesting that the reduction of nephrons from PAE may result in hypertension in later life [60] which may in turn serve as a proximate risk factor for cerebrovascular stroke [61]. Previous research shows that even a single episode of alcohol exposure can result in modifications to chromatin structure, and can induce birth defects [6]. Ethanol interferes with neural stem cell formation and maintenance, which can directly affect neural development [62-64]. Maternal ethanol consumption causes a depletion of neural stem cells, leaving the maturing neural cells vulnerable to genetic damage and resulting in abnormal neural maturation [62-64]. The loss of neural stem cells has been implicated in the etiology of neurodegenerative diseases like Alzheimer’s disease and therapies focused on neural regeneration are a promising experimental approach to minimizing the effects of neurodegeneration [65]. However, the early loss of neural stem cells may pre-dispose individuals with FASD towards an increased risk for dementia. Additionally, PAE has been linked to deficits in hippocampal plasticity as well as impaired memory [66, 67], impacting a brain region already closely associated with Alzheimer’s disease, again suggesting increased risk for dementia [68].

### 1.2 Prenatal nicotine exposure as an initial hit

In the United States, approximately 1 in 14 women reported smoking during pregnancy [69]. Smoking during pregnancy has detrimental effects such as low birthweight, stillbirth, and other birth defects [69]. In rats, nicotine has been demonstrated to induce abnormal brain maturation through the partial inhibition of DNA synthesis in the fetal brain and the elevated presence of ornithine decarboxylase, a biomarker related to cellular maturation [70-72]. Although maternal smoking is associated with adverse outcomes such as learning impairment [70, 73], further studies are necessary to establish the long-term neurological effects of nicotine, specifically. Intrauterine or early life nicotine exposure may contribute towards risk of type 2 diabetes [74]. It has been demonstrated that nicotine exposure *in utero* and in neonate rats leads to a disruption in pancreatic development as well as a reduced quantity and impaired function of beta cells [74, 75]. The elevated levels of beta cell apoptosis in rats as well as changes in glucose homeostasis are associated with type-2 diabetes [74, 75].

### 1.3 Maternal stress and early childhood adversity as an initial hit

A wide variety of factors and diseases, such as depression and preeclampsia, can be considered maternal stressors. Maternal stressors can have consequential effects on the offspring’s long-term health by increasing the risk or prevalence of chronic disease. In the third trimester, it is estimated that approximately 12% of pregnant women experience depression [76]. One study focused on the impact of maternal depression on alterations in DNA methylation in T lymphocytes and the adult hippocampus [77]. This study reported that, at birth, there was predominant chromatin hypomethylation in T lymphocytes isolated from umbilical cord blood [77]. Additionally, this study identified changes in DNA methylation in the hippocampus of adult offspring[77]. This suggests that maternal psychological states may result in life-long and multi-system effects in offspring. Maternal preeclampsia has recently been tied to an increased risk for the later development of cardiovascular and neurological diseases in offspring [78]. In the Helsinki Cohort, offspring born to mothers who developed preeclampsia or gestational hypertension during pregnancy exhibited an increased risk for stroke later in life [78, 79]. Preeclampsia was associated with reduced head circumference [79], whereas gestational hypertension was associated with reduced birth weights and exemplified characteristics pertaining to the ‘brain sparing’ phenomena [79]. Adverse events in early childhood, such as trauma, can increase the likelihood of developing cardiovascular disease as well as type 2 diabetes [80]. Another study using the Helsinki cohort, analyzed the effects of early childhood separation from parents during World War II, when a large number of children were evacuated to Sweden and Denmark [80]. Children who were separated from their parents were twice as likely to be diagnosed with cardiovascular disease in comparison to those who were not [80]. The Adverse Childhood Experiences Study supported a relationship between health risk associated with childhood physical, emotional/sexual abuse, neglect, and household dysfunction [81]. Children were at a higher risk of developing disease in later life when they were exposed to multiple categories of adverse events (e.g. psychological, physical, sexual, household dysfunction) [81]. These exposures were associated in adulthood with an increased incidence of diseases such as ischemic heart disease, cancer, skeletal fractures, and liver disease [81, 82]. The mechanisms that link early adversity to adult-onset disease are unclear. Here, we examine the potential influence of a novel endocrine signal, mediated by a class of circulating, membrane-enclosed structures termed extracellular vesicles.

## 2. Extracellular Vesicles

Membrane-bound extracellular vesicles (EVs) are released from all cell types, contain content derived from the host cell, and have the ability to transfer that content to recipient cells and tissues [83, 84]. EVs are often sub-categorized into two classes, exosomes and ectosomes [85, 86]. Ectosomes are also frequently referred to as shedding vesicles, microvesicles, exosome-like vesicles, nanoparticles, microparticles, and oncosomes [87]. EVs are heterogenous, but potentially functional structures that are carriers of various biomolecules, dependent on the content of their cells-of-origin. They can contain membrane-bound and free proteins including enzymes, whole or fragmented RNAs (mRNA, miRNA, lncRNA, etc.), DNA, and lipids [88-91]. EVs can be taken up by recipient cells through endocytosis or fusion directly with the cell membrane, or function through interactions between EV-bound ligands and cell membrane receptors [92]. Through these interactions, whether transferring its cargo or activating cell receptors, EVs represent a novel endocrine or paracrine mode for intercellular communication [93-96]. Because EVs may mediate both homotypic and heterotypic cell-to-cell communication and even potentially exchange information between distant tissues, they are well-positioned to play both protective and pathogenic roles in normal physiology and in disease states.

## 3. Potential role of EVs as mediators and perpetuators of prenatal and early life insult in diseases of aging

As mentioned earlier, epigenetic modifications of DNA and associated histones as well as alterations in regulatory networks of non-protein-coding RNAs constitute a critical biological substrate in the DOHaD hypothesis (Fig. 1), that transduce the effects of early life experience into both health and disease risks over a lifetime. MicroRNAs (miRNA), a type of small noncoding RNA, that regulate networks of protein-coding genes by translation-inhibition [97, 98] have been localized to EVs. Moreover, miRNAs isolated from circulation and cerebrospinal fluid have been identified as biomarkers for specific diseases and illnesses and may be a potential therapeutic target [99-104]. Cells and tissues from which EVs originate have the potential to control protein translation and function in distal tissues, by transferring RNA and protein cargo in EVs to recipient cells. Consequently, any alteration to the cargo carried by EVs following exposure to an environmental stressor may have immediate and widespread consequences for organ development resulting in a permanent change in developmental trajectory and facilitating an accumulation of damage as an individual ages that later manifests as diseases such as atherosclerosis, dementia, or osteoporosis[105-108]. EVs may have a role in explaining the mechanistic underpinnings of the DOHaD theory and evidence to support this will be discussed here. Currently there is a paucity of research that focuses on the specific question of how EVs mediate progression of diseases of developmental origins. However, in this review, we assess the body of research that exists and reveal connections that supports EVs as mediators, hopefully guiding hypotheses for future work. We have chosen to focus on those diseases for which developmental exposures have been shown to contribute to increased risk of developing.

### 3.1 EVs in the Developmental Origin of Adult-onset Cardiac and Skeletal Disease

#### 3.1.i Cardiovascular disease

In 1990, environmental conditions *in utero* were first documented to have an effect on blood pressure and hypertension in adults [9]. The perturbation-induced discrepancy between fetal and placental size was thought to contribute towards this increased risk for cardiovascular disease [9]. Since these early studies, a number of subsequent studies have linked fetal undernutrition to cardiovascular disease (CVD) [1, 2]. Low birth-weight poses a greater risk for coronary heart disease and stroke in later health, in part due to the reduced rates of fetal growth [13, 109]. The ‘brain-sparing’ phenomena which occurs due to the reduced rates of fetal growth cause a redistribution of blood to favor circulation to the brain. The resulting decrease in systemic blood circulation in arteries may hinder their elasticity[13, 110], and this sequence of events is hypothesized to lead to hypertension and increased susceptibility for hemorrhagic stroke [13, 110]. Additionally, the altered blood circulation affects other organs such as the liver which faces an elevated concentration of plasma fibrinogen [13, 111], a factor associated with thrombotic stroke [13, 112].

One developmental risk factor for CVD later in life is congenital heart disease (CHD), which is one of the most common congenital malformations diagnosed in newborns with a prevalence of 7.5% of live births worldwide [113]. As diagnostics and surgical interventions have improved for CHD, patients are surviving to an older age [114]. In fact, the majority of patients with CHD seen in clinical practice are adults, and they will likely continue to represent the largest proportion of patients requiring life-long medical care [115, 116]. This is because even after surgery in childhood to fix congenital defects, adult CHD patients are at increased rate of CVD risk factors (eg. obesity and hypertension) [117-119] and also in general have an increased risk of CVD (eg. stroke and myocardial infarction) [117, 120-124]. Growing evidence suggests that miRNAs packaged in EVs may contribute to the development of malformations in CHD. A number of studies have identified significantly different levels of circulating miRNAs (miR-34a, miR-142-5p, miR-1275, miR-3664-3p, and miR-4666a-3p) in pregnant women carrying a fetus with CHD compared to women carrying a fetus without CHD [125-127]. Furthermore, bioinformatics analysis has identified these miRNAs as being involved in the regulation of fetal heart development [126-129]. Additionally, prenatal exposure to teratogens, such as alcohol and smoking, may further increase the risk of congenital heart malformations with subsequent lifelong consequences. For example, miR-17-1-3p is increased in EVs from neural stem cells of fetal mice after exposure to ethanol [64] and miR-20a-5p is elevated in EVs isolated from smokers [130]. Both of these miRNAs play a key part in cardiogenesis by participating in the differentiation of progenitor cells in heart muscle. Elevation in their expression is documented to inhibit expression of crucial cardiac progenitor genes like Islet-1 (*Isl1*) and T-box transcription factor 1 (*Tbx1*), resulting in CHD [131].

Another means by which prenatal and early life exposures may contribute to CVD in adulthood is by increasing the predisposition for additional contributory risk factors, such as obesity, insulin resistance, hypertension, abnormal cholesterol, and an increased risk for clotting [55, 132-135]. For example, impaired glucose homeostasis as well as dyslipidemia has been identified in adult animals prenatally exposed to ethanol [55]. Additionally, there is an increased risk of hypertension and obesity in children prenatally exposed to smoking[132], and secondhand smoking in childhood is also associated with subsequent obesity, dyslipidemia, and insulin resistance [133]. Similar outcomes have been attributed to adverse childhood events and maternal stress during pregnancy [134, 135].

This observed phenomenon of fetal and early childhood programming of CVD may be mediated by EVs. For example, cocaine increases release of miR-130a by EVs from human monocyte-derived macrophages and mediates increased smooth muscle proliferation, resulting in pulmonary hypertension [136]. Postnatal oxycodone exposure may also impact vascular smooth muscle cell function by decreasing miR-26a in EVs [137], which can induce a more contractile phenotype in smooth muscle cells [138]. Early exposures like these may predispose an individual to hypertension, by persistently impairing vascular smooth muscle cell function.

An individual may also be primed for dyslipidemia by early life experiences. For instance, ethanol exposure increases miR-145 expression in the fetal mouse brain [139] while oxycodone exposure *in utero* increases miR-128-1-5p and postnatally elevates miR-451-5p in brain-derived EVs from rats [137]. Both miR-145-5p and miR-451a are elevated in EVs derived from senescent platelets obtained from human donors and are identified as being associated with lipid metabolism and vascular disease and inflammation [140]. MiR-128 is enriched in EVs from macrophages stimulated by oxLDL to be pro-atherogenic [141] and is also associated with inflammatory and lipid homeostasis pathways [142]. This means that prenatal and postnatal exposures may promote dyslipidemia inducing EVs from a young age, allowing for accumulation of damage over a longer period of time and increasing risk in an individual.

Additional factors may contribute to this earlier damage to the vasculature and accumulation of atherosclerotic lesions later in life. Ethanol use increases miR-155 [143], which mediates destruction of tight junctions and endothelial barrier via EV transfer [144], potentially impacting fetal vascular development and having lifelong consequences. Smoking decreases EV miR-133a [130], a miRNA that has proven beneficial post hypoxic injuries (eg. myocardial infarction) [145] and its downregulation is associated with development of atherosclerosis and arterial calcification [146], meaning a child exposed *in utero* or via secondhand smoking may suffer from increased risk of atherosclerosis.

Altogether, these first “hits” may ultimately contribute not only to the increased risk for a second “hit”, but also to worse outcomes after a second “hit.” For example, both miR-21 and miR-150 have been shown to promote cellular recovery post-vascular injury via EV transfer [145, 147, 148] and both have been shown to be suppressed by ethanol and smoking, respectively, suggesting that early life exposures have the ability to not only increase the risk for disease in adulthood, but to also worsen the prognosis that results from a secondary insult. This means that even if the effects of prenatal and postnatal exposures are not immediately recognizable, a second “hit” may uncover an underlying condition that worsens recovery.

#### 3.1.ii Osteoporosis and Osteoarthritis

Osteoporosis and osteoarthritis are characterized by tissue remodeling resulting in net loss of bone density and cartilage, respectively. The resulting fractures and debilitating pain decrease mobility and quality of life. Bone and cartilage remodeling occurs through continuous resorption and deposition. This remodeling is controlled by growth factors, hormones, and other regulatory molecules.

During development, EVs within amniotic fluid are rich with growth factors that support angiogenesis [149] and chondrogenesis [150]. In fact, amniotic EVs, upon injection into monoiodoacetate-induced arthritic knee joints *in vivo* restore lost cartilage [150]. Furthermore, EV TGF-beta concentration was significantly positively correlated with cartilage restoration [150]. This means that any perturbations in EV cargo or trafficking that may result from *in utero* exposure has the potential to have negative consequences on proper bone and cartilage formation and integrity, creating a predisposition for osteoporosis and osteoarthritis earlier in life. For example, both PAE and prenatal tobacco smoke (PTS) have been shown in animal models to alter bone and cartilage development [151-154] and reduce birth weight[155], which has been shown to increase risk for osteoporosis and osteoarthritis [156]. These animal studies on PAE and PTS begin to explain some of the underlying mechanisms for the observed higher rates of osteoporosis and osteoarthritis [157, 158]. Moreover, alcohol exposure and smoking impact EV cargo [159, 160], potentially disrupting their normal role in angiogenesis and chondrogenesis [161, 162], processes that are crucial for proper bone and cartilage development *in utero* [163].

Additionally, osteoarthritis is associated with dysfunction of the primary cilia [164, 165], which is one region of the cell membrane that is particularly receptive to growth factors, such as those found in amniotic EVs. Primary cilia serve to directionally orient cell activities [166] and a rich aggregation of transmembrane receptors [167] in this specialized structure facilitates interactions between a cell and its environment to regulate cell growth. Primary cilia regulate bone growth [168, 169], and their loss [170, 171] can result in bone defects [172]. Cholesterol plays an integral role in two key components of primary cilium: cell membrane dynamics and ciliary sonic hedgehog signaling [173-175], and therefore, perturbations in cholesterol biosynthesis can disrupt cilia function, ultimately impacting bone growth and development and contributing to increased risk of osteoporosis and osteoarthritis. Maternal undernutrition [176] and PAE [177] are two of sources of early adversity that can result in dysregulated fetal cholesterol metabolism. Structural bone abnormalities often appear at birth in conditions such as these [178, 179] and in other ciliopathies [180]. To further investigate how cholesterol interferes with primary cilia, 7-dehydrocholesterol reductase (*Dhcr7*) and insulin-induced genes 1 and 2 (*Insig1/2*), genes regulating cholesterol metabolism, were mutated in osteoblasts. The mutant osteoblasts demonstrated dysregulated ciliary EV fusion which was ameliorated by the common cholesterol-reducing drug simvastatin [181]. This reveals that cholesterol metabolism disruption may be one means by which prenatal exposure impairs normal trafficking of EVs, resulting in reduced delivery of crucial growth factors and mediators.

To investigate additional causative mechanisms, mouse chondrocytes were exposed to hypoxic conditions and assessed for primary cilia morphology and expression of hypoxia inducible factors (HIFs). Through knockdown and overexpression paradigms, HIF-2a demonstrated the ability to reduce primary cilia size and count in chondrocytes [165]. This effect may be mediated by EV trafficking, as HIFs have been identified in EVs [182], or by altered EV secretion, as HIFs have also been shown to induce release of EVs [183]. Altogether, this suggests that hypoxic conditions may result in a predisposition for osteoporosis and osteoarthritis. Developmental hypoxia comes in many forms: preeclampsia [184], placental insufficiency [185], gestational diabetes [186], childhood respiratory diseases [187], sleep apnea [188], etc.

In summary, developmental exposures can influence the growth factors and modulators carried by EVs and EV’s interactions with primary cilia, which altogether interferes with bone and cartilage formation and integrity. Developmental exposures such as prenatal alcohol or hypoxia inhibit robust primary cilium formation resulting in poor regenerative capacity of cells and, over time, may result in premature symptomatic diseases of aging such as osteoporosis and osteoarthritis.

### 3.2 EVs in the Developmental Origin of Adult-onset Neurodegeneration

#### 3.2.i Alzheimer’s disease

Alzheimer’s disease (AD) is another aging disease in which environmental factors during early development can result in later adverse health outcomes. As mentioned above, maternal alcohol consumption, causing an abnormal fetal neural maturation and a depletion of fetal neural stem cells during early development, can result in a decreased neural stem cell pool [63]. Consequently, residual stem cell activity in the affected adult may be inadequate to rescue memory deficits or repair dysfunctional synaptic and neural circuits [189-192], potentially increasing the risk while lowering the onset age for dementia and AD in individuals with FASD. Likewise, prenatal stressors during sensitive fetal periods and early environmental factors causing early developmental changes or deficits can have long-lasting effects in an individual, exacerbating AD-like neuropathological changes throughout one’s lifespan [193, 194].

Current experimental evidence supports the hypothesis that amyloid beta (Aβ) plaque aggregation and an imbalance between production and clearance of the plaques and its peptides is an early index and initiating factor in AD [195, 196]. To understand how prenatal stressors can cause neuropathological changes in individuals, an animal study examined the age of onset and development of Aβ plaques throughout the lifespan of male and female mouse offspring of dams prenatally exposed to auditory stress [197]. The authors discovered that, compared to the control mouse group, the prenatal auditory stress (PS) group exhibited earlier onset of beta amyloid pathology, with increased brain deposition of Aβ plaques starting at 2 months. Likewise, PS significantly and persistently increased corticosterone levels in affected offspring compared to control groups, between 2 and 10 months of age and resulted in a persistent neuroendocrine-axis hyperactivity, enduring anxiety-like behavior, and cognitive and motor impairment across age. Overall, prenatal auditory stress significantly accelerated cognitive decline and Aβ plaque aggregation, characteristic of Alzheimer’s disease, with female individuals of the PS group displaying a higher susceptibility for developing AD.

#### 3.2.ii Parkinson’s disease

Parkinson’s disease (PD) is an aging neurologic disease where environmental exposure during prenatal or early developmental period may be risk factors that have a long-term or an extended latency for PD’s pathogenesis in an individual’s later adult life. A possible explanation can be that the decreased population of nigrostriatal dopamine (DA) cell bodies early in life of an individual due to negative environmental exposure may speed up the DA system to reach decreased levels of DA cell bodies associated with PD when combined with normal aging-related loss of the cell bodies later in life [198-200]. It is also possible that the neuronal damage in early development may make an individual more vulnerable to subsequent environmental risk factors to result in PD that may not have occurred without the prior developmental exposure. While dopaminergic neuronal death is a key pathological hallmark of PD [201], it is still unclear whether this pathogenesis is due to events that occurred during crucial development period or during adulthood or a combination of both. An animal study examined the effects of exposure to the environmental neurotoxicants, parquet (PQ) and maneb (MB), during a critical early developmental period and following re-exposure in adulthood [202]. Animals exposed at postnatal day 5-19, equivalent to human infantile period of 2 months to 3 years [203], to PQ or MB alone or in combination exhibited a reduction in tyrosine hydroxylase and dopamine transporter-positive neurons and decreased dopaminergic markers, within the nigrostriatal DA system. Moreover, these decreases were sustained even at 6 months of age, long after the initial exposure, suggesting a permanent inhibition of the DA system. This loss was reflected in progressive decrease in locomotor activity between 6-weeks and 6-months of age, which is consistent with progressive neurotoxicity. In addition to the permanent and progressive effects from early developmental exposures, adult exposure following the developmental exposure produced significantly greater reduction in both locomotor activity and nigral DA neuronal numbers, with increased striatal DA turnover (the ratio of (dihydroxyphenylacetic acid + homovanillic acid)/DA) and its metabolites. It strongly supports the hypothesis that neurotoxicant re-exposure during adulthood enhances the effects of developmental exposures and further disrupts and already vulnerable neural circuit.

Evidence from the above study supports the possibility that the effects of nigrostriatal system damage incurred during the developmental period may only be manifest with age progression and be exacerbated by additional neurotoxicant exposure. These data are consistent with the two-hit hypothesis of DOHaD outlined earlier. The neurotoxic exposure during early developmental period is the initial “hit” that results in permanent neurotoxicity to the DA system. Subsequent neurotoxic exposure or other stressors in adulthood act as the second “hit” that tips the already vulnerable balance to favor development of early PD onset.

#### 3.2.iii Extracellular Vesicles in Alzheimer’s and Parkinson’s Disease

In a prenatally stressed environment, as described above, or in pathological conditions such as Alzheimer’s or Parkinson’s disease, it is possible that EVs act as active mediators in the progression of AD and PD, by spreading the characteristic misfolded proteins like tau and Aβ in AD or like α-synuclein in PD [204, 205].

EVs observed from AD patient brains are enriched with Aβ-oligomers that are known to serve as vectors for inter-neuron transfer, propagating the AD pathology in a prion-like manner [206]. Furthermore, when EV formation and secretion are blocked by downregulating TSG101 and VPS4A proteins *in vitro*, the neuron-to-neuron spread of Aβ-oligomer, and related toxicity was found to also be reduced [206]. Likewise, EVs appear to have the capacity to transfer Aβ to extracellular space, as an *in vitro* study found excessive accumulation of EV marker protein, Alix, within amyloid plaques of AD brain sections while the brain of healthy controls lacked both the amyloid plaques as well as Alix protein [207]. Aβ peptide results from enzymatic cleavage of amyloid precursor protein (APP). The imbalance between Aβ peptide generation and clearance from the brain and its subsequent accumulation in the brain results in the characteristic amyloid plaque formation found in AD patients [208]. In neuronal cell cultures, EVs contain and transport full-length amyloid precursor protein (flAPP), APP metabolites as well as the enzymes required for cleaving flAPP [209, 210]. In addition, EVs can transfer hyper-phosphorylated Tau, considered to be a major neuropathological lesion in AD, from microglia to neurons to spread this tauopathy in the brain of AD patients [211, 212]. Taken together, the evidence for enrichment of Aβ-oligomer and Tau in EVs from AD patient brains, as well as evidence cited previously showing that maternal auditory stress significantly increases Aβ plaques in affected young-adult offspring, supports a hypothesis that EVs play a crucial mediating role in AD pathology, and in transducing the effects of early life experiences into early-onset AD pathology.

EVs have also recently been intensively examined for their role in the progression of Parkinson’s disease in patients [205]. Current evidence supports the hypothesis that α-synuclein (α-Syn) proteins play a critical role in common biochemical pathway important to the pathogenesis of PD [213, 214]. This disease selectively degrades dopaminergic neurons in the substantia nigra pars-compact, while the surviving neurons accumulate Lewy bodies that are composed of fibrillar α-Syn and ubiquitinated proteins [215]. Inflammation and misfolded and aggregated α-Syn proteins are key hallmarks of pathogenesis and progression of PD [213]. Research has shown that injured neurons release EVs containing misfolded α-Syn, which can be taken up by other neurons and by glia, potentially resulting in transcellular spread of misfolded α-Syn and activation of an inflammatory response [214]. It has also been noted that α-Syn itself results in increased EV secretion by murine microglia-type cells, and that these EVs expressed increased Major Histocompatibility Complex (MHC) class II molecules and membrane TNF-α on their membrane surface [216] suggesting that α-Syn-stimulated EVs may also spread inflammation. These EVs significantly increased the apoptosis rate in cortical neurons *in vitro*. Such EV-mediated spread of α-Syn protein may well exacerbate PD by decreasing the number and viability of neighboring healthy neurons while propagating inflammatory mediators to proximate glial cells, to promote concurrent inflammation. Another study observed that introduction of misfolded α-Syn protein in an *in vitro* murine embryonic stem cell model resulted in reduced neural proliferation and decreased expression of neuronal markers while increasing neuronal apoptosis, all accompanied by reduced mRNA and protein levels of Notch-1, Hairy and enhancer of split-5 (Hes-5), and Notch intracellular domain (NICD) [217]. The same study also observed neurogenesis alterations in the hippocampal sub-granular zone in α-Syn transgenic mice with decreased Notch-1, NICD, and Hes-5 expression. It is likely that EVs mediate the transfer of α-Syn and other neuroinflammatory proteins in the brain both during early neurogenesis and in late adulthood to promote PD onset and progression.

Recent studies have also examined the role that EVs play in brain disorders and neurodegeneration due to their capacity to transfer miRNAs that can activate Toll-like receptors (TLRs) and the production of proinflammatory cytokines [218]. Several miRNAs, like miRNA-21, 29a, and let-7, have been documented to bind to TLRs 7-9, and subsequently activate NF-κβ signalling and pro-inflammatory cytokine secretion [219]. While transient inflammation serves an important purpose in assisting the immune system to fight off infectious pathogens, prolonged inflammation during development and adulthood may contribute to neuronal dysfunctions and progression of neurodegenerative diseases such as AD and PD.

### 3.3 EVs in the Developmental Origin of Cancer

The lifetime probability of being diagnosed with cancer ranges from 38-40% and there were, in a predictive model, an estimated 1,806,590 new cases diagnosed in 2020, of which 11,050 were in children (ages 0-14) and 5,800 were in adolescents (ages 15-19) [220]. While there are numerous types of cancer, the underlying pathogenesis is common to all. Genetic mutations and epigenetic reprogramming ultimately result in uncontrolled cellular proliferation and survival [221]. Moreover, EV signaling of these changes can seed tumor growth, promote transformation, protect tumors from immune response, and promote metastasis [222]. Exposures *in utero* and early childhood could be initial triggers of genetic mutations and epigenetic modifications since developmental and cancer biology are so closely linked [223, 224]. Tissue growth and differentiation is regulated by complex cellular processes that involve precise regulation of both cell division and apoptosis during prenatal and postnatal development. EVs have been shown to play a key role in these processes [225]. Because tumorigenesis is intimately linked to the processes of organogenesis, tissue growth and maturation [223], any perturbations during development in EV cargo and subsequent impact on these pathways that control these processes could promote transformation, making teratogens and other developmental insults tumorigenic both in developing cells and into adulthood.

If these mutations and epigenetic reprogramming occur in crucial, highly replicative tissues, then the consequences of *in utero* and childhood exposures may manifest relatively quickly as childhood cancer. One example of an early-onset cancer is neuroblastoma. For instance, evidence from a small case review of 13 children suggests that there are higher rates of neuroblastoma occurrence in children diagnosed with FASD than in the general population (6/13 or 46% vs. 10% in general population) [226]. Gene expression patterns in neuroblastoma are very similar to the expression pattern in neural crest cells, suggesting a common cellular origin, and also suggesting that the embryonic period of neural crest proliferation and migration may be vulnerable to *in utero* exposures which perturb the normal process of organogenesis [227]. PAE has been shown to alter neural crest migration, survival, oxidative stress response, and gene expression pattern [228]. Additionally, as mentioned previously, PAE alters EV cargo [64, 229, 230], potentially perturbing to normal role of stem cell-derived EVs as communicators in development [225] and modulators of oxidative stress response [231]. Ultimately, this phenomenon could at least contribute in part to the higher rate of neuroblastoma observed in children with FASD.

In other instances, the damage that results from accumulated *in utero* or childhood exposures may finally be manifest in adulthood. One well known example is the long-term consequences of diethylstilbestrol (DES) use during pregnancy on offspring, particularly female offspring who are more likely to develop cancers such as clear cell adenocarcinoma [232]. One study showed that DES upregulated specific miRNAs in fetal mouse thymocytes [233]. A number of these miRNAs have been identified in EVs [234] and are associated with cancer development such as breast, liver, brain, prostate, myometrial, and ovarian tumorigenesis [235, 236]. Altogether, this suggests these upregulated miRNAs target a number of proteins that may be involved in cellular survival, apoptosis, cell invasiveness, and oxidative stress common to many tissue types and potentially propagated by EV signaling.[Fig F2-ad-12-6-1516]

**Figure 2. F2-ad-12-6-1516:**
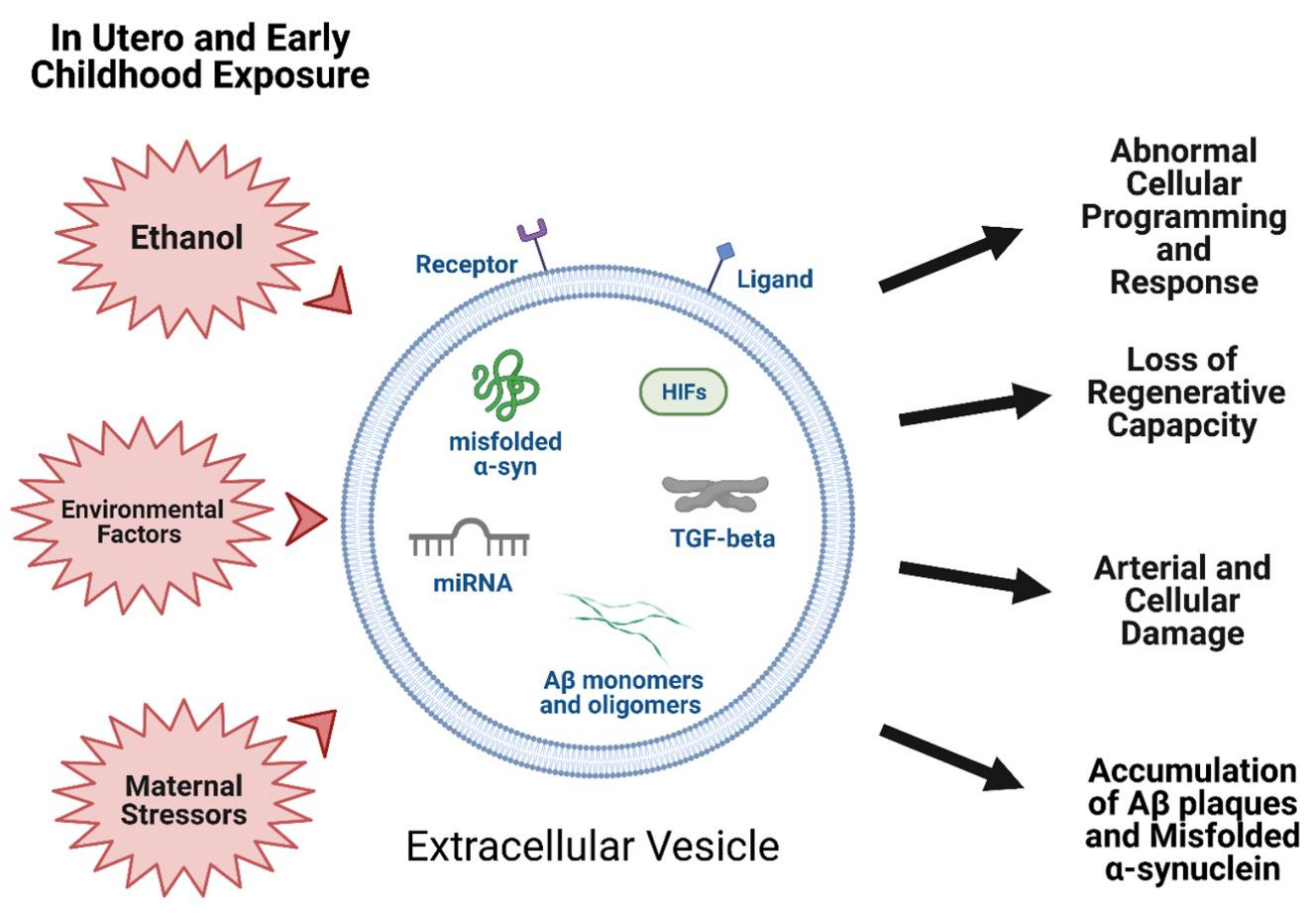
**Early life exposures during pregnancy and childhood have the potential to alter extracellular vesicle composition and cargo that in turn has been shown perturb normal biological processes, contributing to the development of diseases**. (Created with BioRender.com.)

Immune surveillance also plays a critical role in preventing tumor growth and progression. Antigen presenting cells present tumor-specific antigens to effector immune cells (ex. NK cells, T cells) which subsequently target tumor cells for lysis, stopping tumorigenesis [237, 238]. Furthermore, a decrease in lymphocyte population and immunosuppression is associated with an increased risk of cancer development [237, 238]. Prenatal and childhood exposures have been linked to a decrease in immune cell populations and impaired function. For example, PAE impairs immune cell function in offspring [239] and of particular interest for tumorigenesis, it decreases T cell number in the thymus and peripheral blood and impairs T cell response to stimulation of proliferation in rats [240, 241]. These events may be mediated by EVs since they play an important role in immune cell maturation and activation [242-246], including direct and indirect antigen presentation to T cells [247]. Additionally, EVs carry immune modulatory molecules, such as cytokines, costimulatory/inhibitory molecules, growth factors [248-250], that, if increase or decrease in EVs, may alter the expected T cell response to stimuli, affecting immune surveillance effectiveness against tumorigenesis. This is in agreement with the higher rates of cancer observed in individuals with FASD aged 18-44 compared to general population (3.75% FASD vs. 2.0%, general population, 1.9 fold higher) [158]. And this pattern is seen again in PTS exposure and second-hand smoking (SHS) in childhood. One study showed suppressed function of cytotoxic T-cell lymphocytes (CD8+), which resulted in increased successful seeding of lymphoma cells and subsequently more rapid tumor growth in PTS mouse pups [251]. Another study showed similarly suppressed CD8+ function, this time against viral infection [252]. Interestingly, in a rat model of allographic liver transplant, EVs from regulatory T cells were sufficient to decrease CD8+ proliferation and allograft transplant rejection [253], indicating that if EVs are similarly altered following PTS/SHS, they could mediate the suppression of CD8+. This impaired immune cell function could contribute to the observed higher rate of childhood cancers in children exposed to SHS [254] and could increase risk for cancer in early adulthood as more cellular damage accumulates from SHS [255-257].

## 4. Conclusions

While diseases such as CVD, osteoporosis, osteoarthritis, and dementia are typically regarded as diseases of aging, it is important to consider their possible developmental origins. Environmental perturbations during the prenatal period and early childhood may increase risk of these diseases in adulthood and potentially lead to early onset in affected individuals as a result of earlier accumulation of damage. Review of the literature suggests that EVs are possible mediators of the long-term consequences of early life experiences, either directly, by contributing to impaired tissue development, or indirectly, by contributing to the accumulation of additional risk factors like the spread of mis-folded proteins which mediate adult-onset disease. As revealed by our review of the literature, there is a lack of studies focused on the direct question of how EVs mediate progression of diseases of developmental origins. Additional research is needed to more clearly elucidate these mechanisms beyond the relationships we have described above. By gaining a better understanding of how early life influences lead to so many common diseases of the aging, it may be possible to identify those with increased risk at an earlier age. Potential early identification of risk raises the exciting possibility of increased time for interventions to mitigate disease progression allowing for better control of chronic diseases.
